# REAC Noninvasive Neurobiological Stimulation in Autism Spectrum Disorder for Alleviating Stress Impact

**DOI:** 10.1007/s41252-022-00293-3

**Published:** 2022-10-01

**Authors:** Arianna Rinaldi, Márcia C. Marins Martins, Margherita Maioli, Salvatore Rinaldi, Vania Fontani

**Affiliations:** 1 Department of Biomedical Sciences, University of Sassari, Sassari, Italy; 2 Department of Adaptive Neuro Psycho Physio Pathology and Neuro Psycho Physical Optimization, Rinaldi Fontani Institute, Florence, Italy; 3 International Scientific Society of Neuro Psycho Physical Optimization With REAC Technology, Brazilian Branch, São Paulo, Brazil; 4 Research Department, Rinaldi Fontani Foundation, Florence, Italy

**Keywords:** Autism, Depression, Anxiety, Allostatic overload, REAC, Neurobiological stimulation

## Abstract

**Objectives:**

Autism spectrum disorder (ASD) symptoms can become more evident because of different factors. Among these, depression, anxiety, and stress play an important role. Additionally, several studies have revealed the impact of the COVID-19 pandemic on participants with ASD. In previous studies, two noninvasive neurobiological stimulation treatments with radio electric asymmetric conveyer (REAC) technology, called neuropostural optimization (NPO) and neuropsychophysical optimization (NPPO), were shown to be effective in improving the subjective response to environmental stressors in the general population and in ASD population. Based on the proven efficacy of REAC NPO and NPPOs treatments in alleviating anxiety, stress, and depression, the purpose of this study is to verify how these treatments can reduce the severity of ASD symptoms expression, which is aggravated by depression, anxiety, and stress. The treatments’ effects were perceived by caregivers and assessed by the Autism Treatment Evaluation Checklist (ATEC).

**Methods:**

This study involved 46 children with a previous diagnosis of ASD made using the Autism Diagnostic Observation Schedule and Autism Diagnostic Interview-Revised. The participants received one session of NPO treatment and one NPPOs treatment cycle of 18 sessions, administered within approximately 3 weeks. The Autism Treatment Evaluation Checklist (ATEC) was used to evaluate the efficacy of the REAC treatments. ATEC allows to evaluate four clusters (speech or language communication; sociability; sensory or cognitive awareness; and health/physical/behavior) through a numerical scale that measures increasing levels of ASD severity.

**Results:**

The comparison between the scores of the ATEC administered pre- and post-REAC treatments highlighted an improvement of ASD symptoms in each of the four clusters of ATEC.

**Conclusions:**

The results confirm the usefulness of REAC treatments to optimize the individual response to environmental stressors and reduce the symptomatic expression and deficits present in ASD.

Autism spectrum disorders (ASD) are characterized by a generalized impairment in various areas of development, such as social interaction skills, verbal and nonverbal communication, social and emotional reciprocity, adequate creation and maintenance of social ties, and areas of interests and activities (American Psychiatric Association, 2013). These disorders also manifest through stereotypical use of movement, language, or objects, excessive adherence to routine, motor or verbal rituals or resistance to change, fixation for special or restricted interests in abnormal duration or intensity, and hyper- or hypo-reactivity to sensory stimuli or unusual interest in particular details of the environment (American Psychiatric Association, 2013). Depression, anxiety, and stress (DAS) emerge in the foreground among the causes that can aggravate the typical ASD symptoms (Hollocks et al., 2019; Uljarevic et al., 2020), and they can impact also on atypical autonomic dysregulations (Taylor et al., 2021). DAS seemed to be ubiquitous during the COVID-19 pandemic in both healthy participants and those who contracted the virus and manifested postinfectious syndrome (Bueno-Notivol et al., 2021; Cenat et al., 2021). Several studies have shown that the impact of the COVID-19 pandemic was more evident in participants with ASD (Mutluer et al., 2020) than in non-ASD controls (Amorim et al., 2020), because of the difficulty of participants with ASD to adapt to environmental mutations.

In this study, we used two noninvasive neurobiological stimulation treatments with the radio electric asymmetric conveyer (REAC) technology, called neuropostural optimization (Rinaldi et al., 2011) (NPO) and neuropsychophysical optimization (NPPO), sequentially administered. NPO and NPPOs treatments have been shown to be effective in various stress-related disorders and symptoms (Fontani et al., 2011; Rinaldi et al., 2010), anxiety, and depression (Olivieri et al., 2011; Rinaldi et al., 2019), even during the COVID-19 pandemic (Pinheiro Barcessat et al., 2020a, 2020b). This evidence of the efficacy of REAC NPO and NPPOs in reducing symptomatic expressions of depression, anxiety, and stress led us to verify how these neurobiological stimulation treatments could improve the symptomatic expression and deficits present in ASD, potentially aggravated by the COVID-19 pandemic.

The multiplicity of symptoms or deficits present in ASD has complicated the evaluation of the effectiveness of various treatments administered to participants with ASDs. A popular test to evaluate the effectiveness of various types of treatments is the Autism Treatment Evaluation Checklist (ATEC) (Magiati et al., 2011; Mahapatra et al., 2018). It is an agile, fast, and sensitive tool that allows an immediate and intelligible comparison between evaluations expressed in numerical scale, detected before and after treatment by ASD caregivers. For these reasons, in this study, we used the ATEC to evaluate the efficacy of the REAC NPO and NPPO neurobiological stimulation treatments administered to participants with ASD during the COVID-19 pandemic.

## Methods

### Participants

The study population consists exclusively of children with a previous diagnosis of ASD made in other centers using the Autism Diagnostic Observation Schedule (ADOS) and the Autism Diagnostic Interview-Revised (ADI-R). A total of 46 children spontaneously brought by their parents to our clinics to be treated with REAC neuromodulation treatments from April to December 2020 participated in this study. No inclusion or exclusion criteria were applied. There were no cases of Asperger’s syndrome or pervasive development disorder in the population, and all 46 children presented symptoms of classic autism disorder. None of them was taking any drug therapy. There were 12 females (average age of 9.00 ± 4) and 34 males (average age of 10.15 ± 4.57). The global average age was 9.85 ± 4.42.

### Procedure

At T0, the caregivers were administered the ATEC checklist. At T1, the subjects were administered the NPO treatment, consisting of only one session. One hour later, at T2, they received the first session of the NPPOs treatment. In the following days, they were administered the other NPPOs treatment sessions, concluding with the last eighteenth one within about 3 weeks from the start of the treatment. Approximately 3 months after the end of the NPPOs treatment, at T3, the caregivers compiled the second ATEC for the follow-up.

### Measures

#### Autism Treatment Evaluation Checklist (ATEC)

The ATEC is a form designed to be compiled by parents, teachers, or caregivers (Geier et al., 2013) and consists of 77 items divided into 4 subtests. The first assesses speech or language communication and comprises 14 items: score range, 0–28. The second assesses sociability with 20 items and has a score range of 0–40. The third assesses sensory or cognitive awareness with 18 items and has a score range of 0–36. The fourth assesses the health/physical/behavior with 25 items and has a score range of 0–75. The rating scale is divided into 10 centiles with an increasing level of ASD severity, where the first centile, 0–9 corresponds to a score range of 0–30, and the tenth centile, 90–99 corresponds to a score range of 104–179. Parents compiled the first ATEC before starting the REAC NPO treatment and the NPPO/NPPO-CB treatment cycle; they compiled the second ATEC at the follow-up approximately 3 months after treatment.

#### REAC Technology

The REAC technology was designed for neurobiological stimulation treatments through an optimization of the endogenous bioelectric activity (EBA) fundamental to guarantee the best neurotransmission processes (Levin, 2014). The REAC technology devices used in this study were BENE mod. 110 (ASMED, Florence, Italy). In the scientific literature and the postmarket surveillance of REAC devices, no side effects have been reported.

#### REAC Neuropostural Optimization Treatment

REAC NPO (Rinaldi et al., 2011), the first noninvasive treatment to be administered, aims to check the subject’s responsiveness to neuromodulation. It is a preprogrammed single session treatment, and it is administered by applying the tip of the metallic REAC asymmetric conveyer probe (ACP) to a specific area of the ear, according to the NPO protocol. Previous studies have shown that NPO treatment is able to induce an overall reorganization of postural and motor controls (Fontani et al., 2022; Mura et al., 2012; Rinaldi et al., 2014). This capacity of the NPO treatment was proved even in neurodegenerative diseases such as Parkinson’s (Fontani et al., 2012a, 2012b) and Alzheimer’s (Olazaran et al., 2013, 2014).

#### REAC Neuro-Psycho-Physical Optimization Treatments

NPPO neurobiological stimulation treatments comprise an 18-sessions cycle. These treatments aim to improve the psychophysical response to current environmental factors and the reorganization of the previous altered responses to exposome (Robinson & Vrijheid, 2015; Wild, 2005) at cognitive and behavioral levels (Fontani et al., 2012a, 2012b; Rinaldi et al., 2019). The NPPOs treatments are preprogrammed and can be administered via two modalities (Pinheiro Barcessat et al., 2020a), with a punctiform ACP on the auricle or with a planar ACP in the cervico-brachial region. Both modalities are noninvasive and equivalent in efficacy (Pinheiro Barcessat et al., 2020b), and offer the most tolerable option to young patients, according to their level of cooperation. Each session lasts approximately 30 s to 4 min, depending on the administration modality; the minimum time interval between sessions is 1 h. The maximum number of sessions per day is usually four. The number of cycles administered depends on the patient’s condition. The patients investigated in this study received one treatment cycle and administered within approximately 3 weeks.

### Data Analyses

The statistical analysis was performed using the Statistical Package for Social Science (SPSS), version 22 (SPSS Inc., Chicago, IL, USA). For this study, the distributions of subtest results in pretreatment (T0) and posttreatment (T3) were evaluated with the Wilcoxon and the signed-rank test, assuming a *p* value < 0.05 as statistically significant.

## Results

### ATEC Scores

The scores of each of the four subtests of the ATEC administered pretreatment were calculated and compared with the scores obtained in the posttreatment ATEC administration. The data are presented with box plots, where the box height indicates the values within which 50% of the measured values are contained, whereas the length of the whiskers represents the tails of the distribution: the greater the length, the greater the dispersion of values below the first quartile or above the third quartile. Tukey’s fences method was used to isolate potential outliers. It is a nonparametric method developed to detect observations out of the normal range by using interquartile ranges (IQRs). The box height represents the IQR and indicates the distance between the first and third quartiles (Q1–Q3). The ranges between Q2–k (Q3 − Q1) and Q2 + k (Q3 − Q1) are referred to as fences. Data points below the lower fence or above the higher fence are identified as outliers. The value *k* determines the width of the fences. The larger the value of *k*, the lower the number of outliers that will be detected. In our analyses, we set *k* = 1.5.

### Speech/Language Communication

**Fig. 1:**
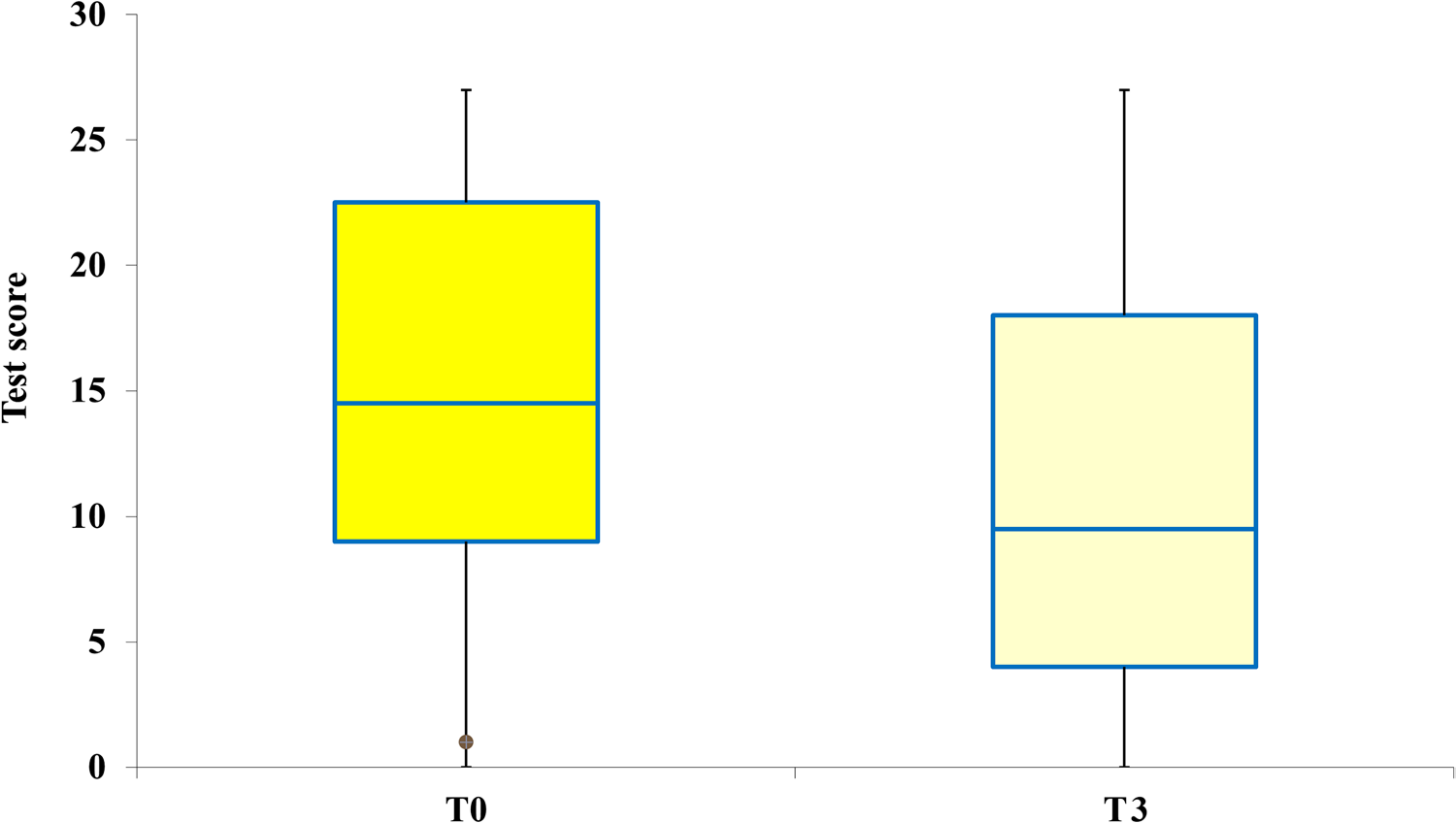
Distribution of the ATEC scores before and after the NPPOs treatment and the shift of the median ATEC value from the sixth to the fourth centile

For the subtest speech/language communication, the comparison of the scores showed that a single REAC NPO and NPPOs treatment cycle was effective in 69.57% of participants with ASD, with *p* < 0.005. This implies that in 32 participants, the parents or caregivers appreciated an improvement in their children with ASD, whereas, in 14 participants, a single treatment cycle was not sufficient to determine an improvement perceivable by parents or caregivers. In particular, the median ATEC value moves from the sixth to the fourth centile, as shown in Fig. 1.

### Sociability

**Fig. 2:**
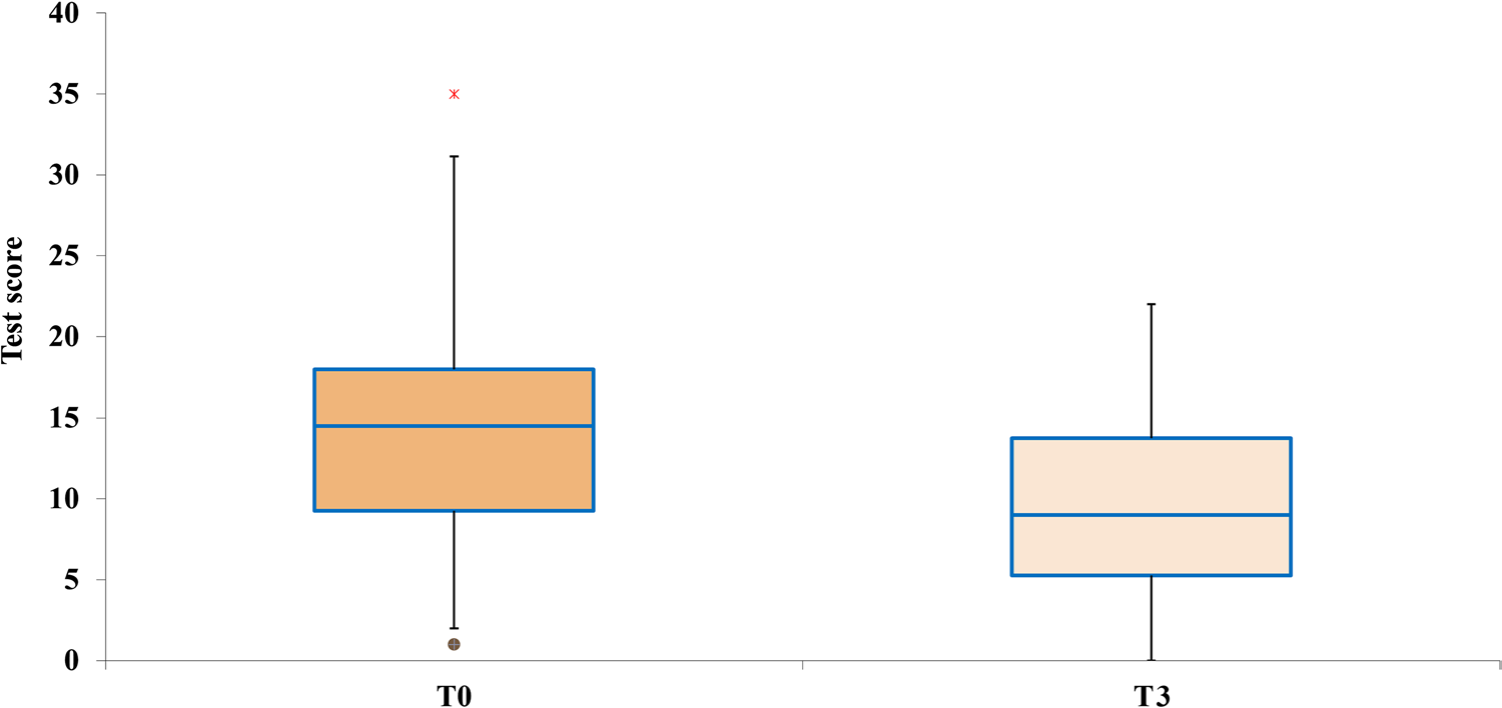
Distribution of the ATEC scores before and after the NPPOs treatment and the shift of the median ATEC value from the sixth to the third centile

For the subtest sociability, a single REAC NPO and NPPOs treatment cycle was effective in 84.78% of participants with ASD, with *p* < 0.005. This implies that in 39 participants, the parents or caregivers appreciated an improvement in their children with ASD, whereas, in 7 participants, a single treatment cycle was not sufficient to determine an improvement perceivable by parents or caregivers. In particular, the median ATEC value moves from the sixth to the third centile, as shown in Fig. 2.

### Sensory/Cognitive Awareness

**Fig. 3:**
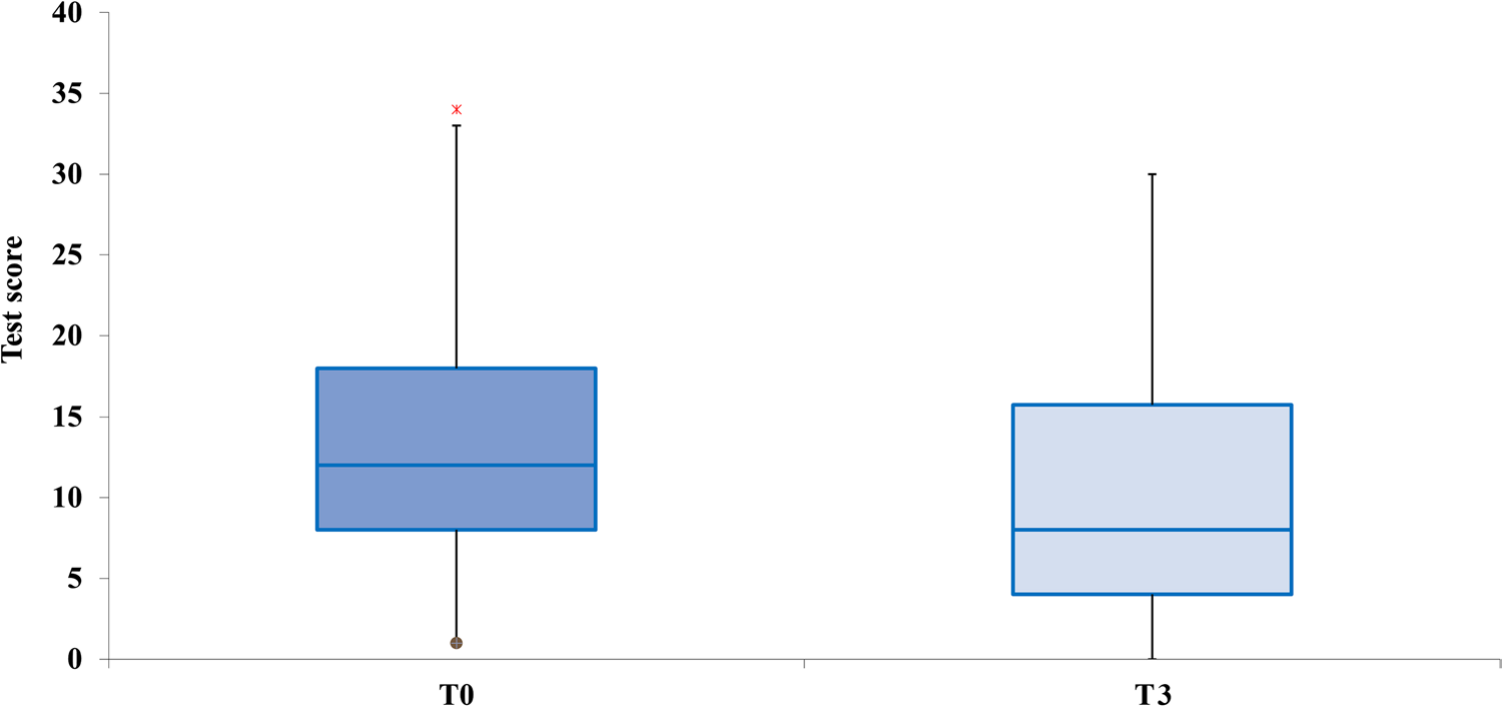
Distribution of the ATEC scores before and after the NPPOs treatment and the shift of the median ATEC value from the fourth to the second centile

For the subtest sensory/cognitive awareness, a single REAC NPO and NPPOs treatment cycle was effective in 78.26% of participants with ASD, with *p* < 0.005. This implies that in 36 participants, the parents or caregivers appreciated an improvement in their children with ASD, whereas, in 10 participants, a single treatment cycle was not sufficient to determine an improvement perceivable by parents or caregivers. In particular, the median ATEC value moves from the fourth to the second centile, as shown in Fig. 3.

### Health/Physical/Behavior

**Fig. 4:**
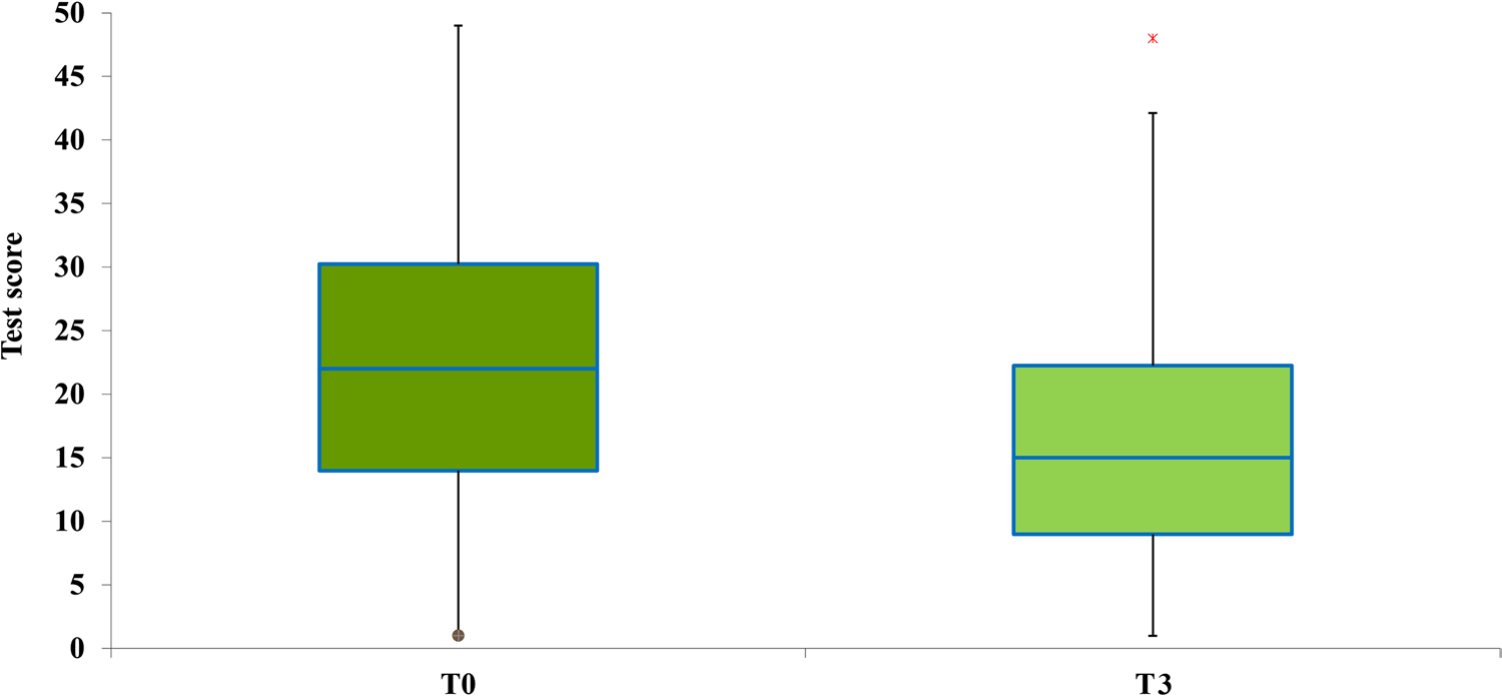
Distribution of the ATEC scores before and after the NPPOs treatment and the shift of the median ATEC value from the sixth to the third centile

For the subtest health/physical/behavior, a single REAC NPO and NPPOs treatment cycle was effective in 89.13% of participants with ASD, with *p* < 0.005. This implies that in 41 participants, the parents or caregivers appreciated an improvement in their children with ASD, whereas, in 5 participants, a single treatment cycle was not sufficient to determine an improvement perceivable by parents or caregivers. In particular, the median ATEC value moves from the sixth to the third centile, as shown in Fig. 4.

### ATEC Global Score Analysis

**Fig. 5:**
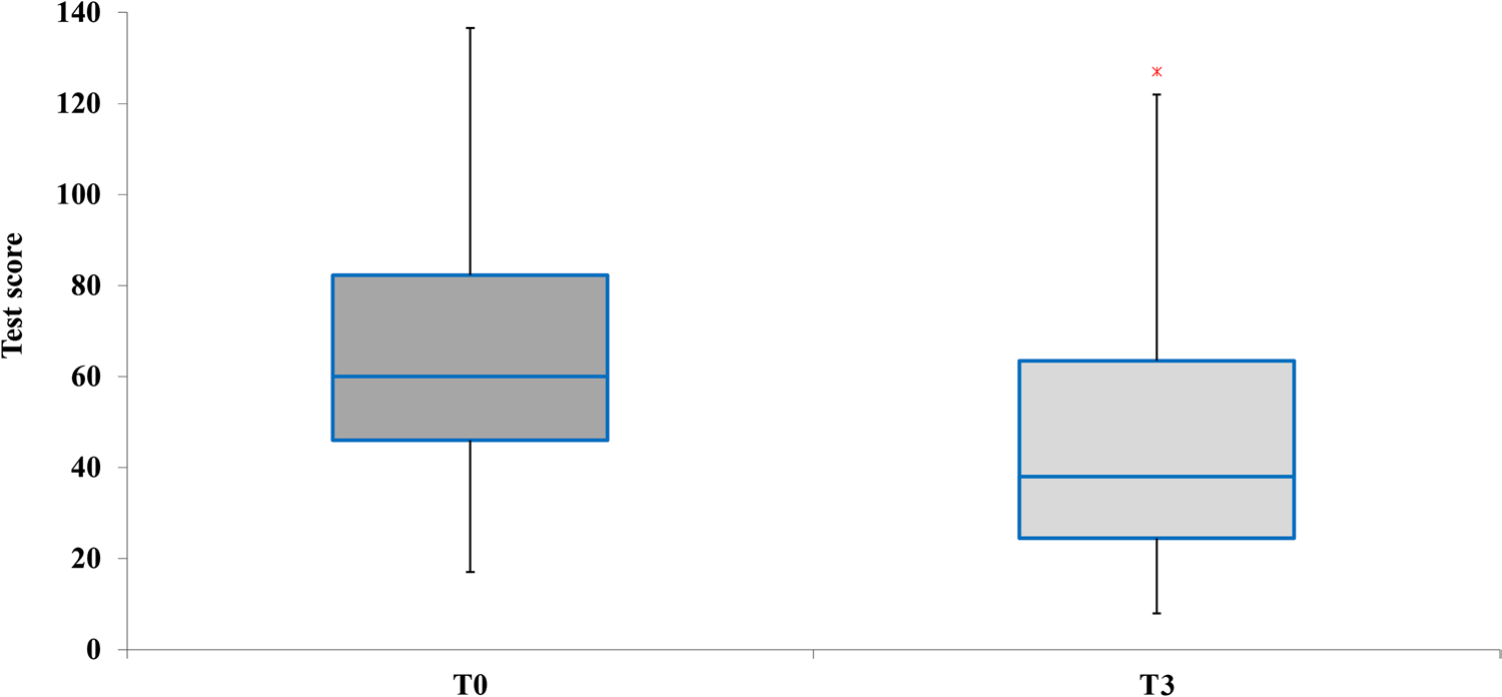
Distribution of the ATEC global scores before and after the NPPOs treatment and the shift of the global average score from the seventh to fifth centile

Analysis of the ATEC global score given by the sum of the subtests and comparison of the results between pre- and post-REAC NPO and NPPOs treatment cycles reveal that the treatment was effective in 45 of the 46 participants (98.83%); their scores improved, with *p* < 0.005. Moreover, after REAC NPO and NPPOs treatments, the global average score declines from the seventh to fifth centile on the ATEC rating scale from 64.17 to 45.54, as shown in Fig. 5.

## Discussion

Various combinations of environmental stressors have been expressed in the literature via numerous terminologies, such as the term *distress*, whose Greek suffix “dis” underlines the dysfunctionality of the stress response. Sterling and Eyer, (1988) coined the term *allostatic overload*, which indicates that an organism can no longer functionally respond to environmental stressors, predisposing it to pathological conditions. More recently, Wild, (2005) introduced the term *exposome*, indicating environmental exposures throughout life, including lifestyle factors, from the prenatal period onward. Regardless of how the summation of environmental stressors is defined, they affect human behavior, not only at the cognitive and relational levels but also at the molecular level, inducing initially invisible changes such as epigenetic ones (Plusquin et al., 2019).

Vulnerability to stressors is particularly observable in ASD. The stress response in children with ASD may be triggered even by minor situations and last longer than in neurotypical children (Corbett et al., 2012; Spratt et al., 2012), increasing depression and anxiety (Nimmo-Smith et al., 2020), which are often present with ASD. In fact, data from the onset of the COVID-19 pandemic revealed that depression affects an estimated 7% of children and 26% of adults with autism (Croen et al., 2015; Greenlee et al., 2016), whereas anxiety disorders affect an estimated 11–42% of people with autism (Romero et al., 2016; Vasa et al., 2016). As for stress, although no study has yet quantified its presence as a percentage in the autistic population, many studies have emphasized its role in amplifying the deficits and symptoms present in ASD (Baron et al., 2006; Fuld, 2018). As shown in Fig. 5, the overall results demonstrate how REAC NPO and NPPOs neurobiological stimulation treatments can induce a better neuropsychophysical response to environmental factors. This global improvement is expressed through a reduction of deficits and symptoms typical of ASD and can be detected by comparing pre- and posttreatment ATEC results. Comparing the global average score before and after REAC NPO and NPPOs treatments, we observe that the posttreatment score declined from the fifth to the third centile on the ATEC rating scale, dropping from 64.17 to 45.54.

### Limitations and Future Research

Caution is advisable in interpreting the parents/caregivers’ scoring of ATEC given that they were aware of the treatment, but we must consider as well that the parents/caregivers’ awareness and the load of their expectations can weigh in an ambivalent way on their sensitivity in evaluating if and how their children benefit from a specific treatment. Yet, the ATEC is a very sensitive tool specially designed to measure the effectiveness of therapeutic interventions by evaluating highly individualized changes that can best be caught thanks to the personal sensitivity of parents and caregivers.

The overall improvement resulting from the ATEC can be explained as the REAC treatments capacity of globally reorganizing the brain pattern activation in terms of greater efficiency, specificity, and competence of the activated areas (Rinaldi et al., 2014). This neuromodulation effect allows NPPOs treatments to optimize the individual response to environmental stressors and face the neuropsychological and behavioral effects of the exposome pressure and the allostatic overload induced by the COVID-19 pandemic in participants with ASD. Further research is desirable to confirm the results obtained in increasingly large populations.

## Data Availability

All data are available at the Open Science Framework (https://osf.io/vywzp/).
